# WTAP Is Correlated With Unfavorable Prognosis, Tumor Cell Proliferation, and Immune Infiltration in Hepatocellular Carcinoma

**DOI:** 10.3389/fonc.2022.852000

**Published:** 2022-04-11

**Authors:** Linjun Liang, Hongfa Xu, Qichao Dong, Lige Qiu, Ligong Lu, Qing Yang, Wei Zhao, Yong Li

**Affiliations:** ^1^ Zhuhai Precision Medical Center, Zhuhai Hospital Affiliated With Jinan University (Zhuhai People’s Hospital), Zhuhai, China; ^2^ Guangdong Provincial Key Laboratory of Tumor Interventional Diagnosis and Treatment, Zhuhai People’s Hospital (Zhuhai Hospital Affiliated With Jinan University), Zhuhai, China; ^3^ Department of Oncology, The Affiliated Hospital of Guangdong Medical University, Zhanjiang, China; ^4^ Department of General Surgery, Zhuhai People’s Hospital (Zhuhai Hospital Affiliated With Jinan University), Zhuhai, China; ^5^ Department of Infectious Diseases and Hepatology, Zhuhai People’s Hospital (Zhuhai Hospital Affiliated With Jinan University), Zhuhai, China

**Keywords:** hepatocellular carcinoma, WTAP, immune infiltration, oncogenes, N6-methyladenosine

## Abstract

WTAP is involved in various pathological and physiological processes, but its function in hepatocellular carcinoma (HCC) remains elusive. In this study, we investigated the role of WTAP in HCC. Firstly, the mRNA and protein of WTAP were expressed highly in HCC tissue, which reflected clinicopathological characteristics of HCC patients. Then, an interactive analysis of genetic profiles and Kaplan–Meier curves was performed to show that WTAP was an independent predictor of survival of HCC patients. Meanwhile, genes co-expressed with WTAP, potential protein–protein interactions, related signaling pathways, and immune cell infiltration were identified. It was found that high WTAP expression correlated with enhanced interactions between cytokines and their receptors, cell cycle, and chemokine signaling pathways, as well as increased immune cell infiltration. At last, WTAP knockdown experiments *in vitro* indicate that the WTAP silencing inhibited HCC proliferation and aggressiveness. We conclude that WTAP may be a novel biomarker for prognosis and a therapeutic target for HCC.

## Introduction

Hepatocellular carcinoma (HCC), with a strong tendency to metastasize, is a common hepatobiliary cancer. HCC has high rates of morbidity and mortality, accounting for 8.2% of all cancer-related deaths (1). Although considerable advancements in HCC management have been made, patient prognosis remains abysmal (2). The short overall survival of patients is often attributed to the insidious onset of HCC, the difficulty for radical resection, and chemotherapy resistance. Moreover, the unclear molecular mechanisms of HCC have made the situation worse. Aberrant gene expression may drive tumorigenesis and be an indicator of patient prognosis. Further studies elucidating the potential molecular mechanisms to develop novel, more targeted treatments for HCC are needed to reduce patient mortality.

Increasing evidence shows that epigenetic dysregulation plays an important role in the genesis and progression of malignancy. N6-methyladenosine modification (m6A), a highly evolutionarily conserved molecule, is the most common means of eukaryotic mRNA modification that makes up 0.1–0.4% of adenosine nucleotides (3). The modification of m6A is a dynamic process regulated by m6A demethylases and methylases, which are called “writers” (methyltransferases), “erasers” (demethylases), and “readers” (effector proteins) (4). m6A “writers” include METTL3, METTL14, WTAP, KIAA1429, and RBM15 (5). On the contrary, m6A “erasers” FTO and ALKBH5 mediate demethylation (6, 7). Furthermore, the YTH protein family is severs as effector proteins (8, 9).

The deregulation of m6A modification is frequently catalyzed by tumor suppressor genes or oncogenes across several types of cancers, including HCC (10). Available literature highlights m6A modification and m6A regulators as important components of signal transduction, cancer stem cell formation, cancer metabolism, and epithelial–mesenchymal transition (11). For example, over expression of METLL3 and FTO are associated with tumor metastasis both *in vitro* and *in vivo*, which also predicts poor prognosis (12, 13). Moreover, METLL4 expression is downregulated and predicts poor recurrence-free survival in HCC and associates with tumor metastasis both *in vitro* and *in vivo* (14).

As the classical complex of “writers”, WTAP has been recently shown to promote tumor progression in an m6A-dependent manner. WTAP is strongly expressed and associated to an unfavorable prognosis in cancers such as osteosarcoma, glioma, and acute myeloid leukemia (15–17). It also contributes to the several aggressive features of various cancers. For instance, WTAP enhances the proliferation of renal cell carcinoma by stabilizing *CDK2* mRNA expression (18) and the aggressiveness of cholangiocarcinoma by promoting MMP7 and MMP28, both of which represent metastasis markers (19). Although multiple lines of evidence indicate that WTAP is likely an oncogene, there is limited evidence on the role of WTAP in HCC (20).

In this study, we aimed to investigate the effects of WTAP on HCC. Expression levels of *WTAP* mRNA between HCC and normal liver samples and its relationship with clinical prognosis was investigate firstly. Then, we assessed co-expressed genes, protein–protein interaction networks, and its effect on the degree of immune cell infiltration. Moreover, we knocked down WTAP to explore its effect on tumor cell proliferation and invasion. Thus, we infer that WTAP could serve as a prognostic marker for HCC patients.

## Materials and Methods

### Data Processing and Analysis

The mRNA level of *WTAP* in HCC was analyzed using the Tumor Immune Estimation Resource (TIMER) database (https://cistrome.shinyapps.io/timer/) and Genomic Data Commons (https://portal.gdc.cancer.gov/). Data of 474 RNA sequences and relevant clinical information of patients with HCC were retrieved from the HCC project of TCGA. Unavailable or unknown clinical data were considered a missing value.

Tumor samples were further stratified into a high or low group in accordance with the median *WATP* mRNA expression level. All RNA-sequencing (RNA-Seq) data were converted to the transcripts per million reads format. After log2 transformation, two sets of *t*-test were implemented, and statistical significance was fixed at *P* < 0.05. Data analysis was assisted by the R program (version 3.6.3), and the R package “ggplot2” was used to draw box plots.

### Tissue Microarrays Analysis

Tissue microarrays were purchased from Chaoxing Biotechnology (Shanghai, China). The tissue microarrays contained 180 samples including liver tumor and healthy adjacent tissues. All samples had detailed clinical information including sex, prognosis, age, and grade. Immunohistochemical staining was carried out in strict compliance to instructions stipulated by the manufacturer. Nuclear and cytoplasmic staining intensities were graded based on the following scale: 0, no staining; 1, weak; 2, medium; 3, strong. The level of staining was graded from 0% to 100%. Scores of 160 or lower indicated a low WTAP level and scores of 161 or higher indicated a high WTAP level.

### Immunohistochemistry Analysis

Immunohistochemistry images of WTAP expression in HCC normal tissues and tumor tissues were gathered on the website The Human Protein Atlas (http://www.proteinatlas.org/) and analyzed.

### Overall Survival Analysis

According to the median expression of WTAP, all participants were stratified according to low or high WTAP expression. To assess the impact of WTAP expression on the clinical outcomes of patients with HCC, we constructed a prognostic classifier using Kaplan-Meier survival curves and compared the survival disparities using R packages “survminer” and “sunival”. To verify the results, we further constructed Kaplan-Meier survival curves using the data extracted from the TIMER and Gene Expression Profiling Interactive Analysis databases (http://gepia.cancer-pku.cn/).

### Regression Analysis

Univariate logistic regression analysis to delineate whether the WTAP affected the overall survival of patients in the two cohorts has been performed. A multivariate Cox regression analysis was utilized to determine whether WTAP is an independent prognostic factor for the survival of patients with HCC. Results with *P* < 0.05 were considered as statistically significant. Further, a receiver operating characteristic curve generated using the pROC package was also utilized to assess whether WTAP has a prognostic value in patients with HCC.

### Co-Expression Analysis

To screen for proteins interacting with WTAP, genes that were co-expressed with WTAP were verified by the cBioPortal database (https://www.cbioportal.org/), and the top 50 of genes are shown in a heatmap. The top 10 significant genes that correlated with WTAP were subjected to additional analysis using the TIMER database to determine if they were related to WTAP expression.

### Comprehensive PPI Analysis

PPI analysis was carried out by GeneMANIA (http://genemania.org/), and the results were confirmed by The Search Tool for the Retrieval of Interacting Genes/Proteins website (https://string-db.org/). By importing the WTAP data into this online tool, we obtained the PPI network information and collected the top 10 interacting proteins. The confidence scores of these proteins were >0.9. Scores of >0.7 were considered significant.

### Gene Ontology and Kyoto Encyclopedia of Genes and Genomes Pathway Enrichment Analyses

After identifying the genes co-expressed with WTAP using the cBioPortal database, we imported them into the online analysis tool Hiplot (https://hiplot.com.cn) and performed enrichment analyses in terms of cellular components, molecular functions, biological processes, and Kyoto Encyclopedia of Genes and Genomes (KEGG).

### Relationship Between WTAP Expression and Immunity

Correlation analysis of WTAP expression level and immune-infiltrating cells was using the TIMER database. Using the “GSVA” package of R software, Single-Sample Gene Set Enrichment Analysis (ssGSEA) was performed. The co-expression analysis of WTAP and immune-related genes also have been performed, including genes encoding immune checkpoints, major histocompatibility complexes, chemokines, chemokine receptors, and immune activation and immunosuppression molecules.

### Cell Culture and siRNA Transfection

The human HCC cell lines used in this study were stored in Zhuhai Precision Medical Center. In a 37°C incubator containing 5% CO_2_, MHCC97-H and SMCC-7721 were incubated in Dulbecco’s modified Eagle medium (DMEM; Gibco, MA, USA) or RPMI 1640 (Gibco, MA, USA) that contained 10% fetal bovine serum. The HCC cells were transfected with siRNA (RiboBio, Guangzhou, China) using Lipofectamine 3000 Reagent (Invitrogen, CA, USA) to produce a working concentration of 100 nM, following to the protocols of the manufacturer. The sequence of siRNAs used is as follows: siWTAP#1:5’-GGAACAGACTAAAGACAAA-3’; and siWTAP#2:5’-CTAAGAGAGTCTGAAGAAA-3’.

### Quantitative Real-Time Polymerase Chain Reaction Analysis

The RNA-Quick Purification Kit (ES Science, Shanghai, China) was used to isolate the RNA from cells. The Revert Aid First Strand cDNA Synthesis Kit (Thermo, MA, USA) was used to obtain cDNA from RNA. SYBR Green Master Mix II was used for RT-qPCR analysis, with *GAPDH* as the internal control. The 2^-ΔΔCT^ approach was used to analyze the data. The sequence of the PCR primer used was as follows: WTAP-forward: 5’-GCAACAACAGCAGGAGTCTGCA-3’, WTAP-reverse: 5’-CTGCTGGACTTGCTTGAGGTAC-3’.

### MTS Proliferation Assay

In 96-well plates, 800 cells per well were incubated with the indicated reagents. Thereafter, 20 µL CellTiter 96 AQueous One Solution (Promega, WI, USA) was added to each well. The mixture was incubated for 3 h. Optical density was measured at 490 nm.

### Colony Formation Assay

500 treated cells were coated into 6-well plates with three repetitions. After incubation for 14 days, these plates were washed with PBS twice, fixed by 4% polymethanol for 10 min and stained with 0.1% crystal violet solution within 10 min. A cluster of 50 cells was defined as a colony.

### Transwell Assay

To examine cell migration, 1 × 10^5^ treated cells mixed in 100 µL of serum-free DMEM or 1640 medium were placed in the upper Transwell chamber. Thereafter, 500 µL of the complete culture medium was placed in the lower chambers. After incubating for 24 h, cells that migrated were fixed for 30 min with 4% paraformaldehyde and subjected to 20 min of 0.1% crystal violet staining, whereas non-migrated cells were remove with a cotton swab. The wells were then washed with water and air-dried before quantifying the total migrated cells.

### Statistical Analysis

Data were analyzed using SPSS 23.0 and R version 3.6.1. Data are presented as mean ± SD. Intergroup differences were established using independent and paired sample *t*-tests. Logistic and Cox regression models were used to analyze the relationship between WTAP expression and clinical characteristics. Statistical significance was set at *P* < 0.05.

## Results

### WATP Is More Highly Expressed in HCC Samples

**Figure 1:**
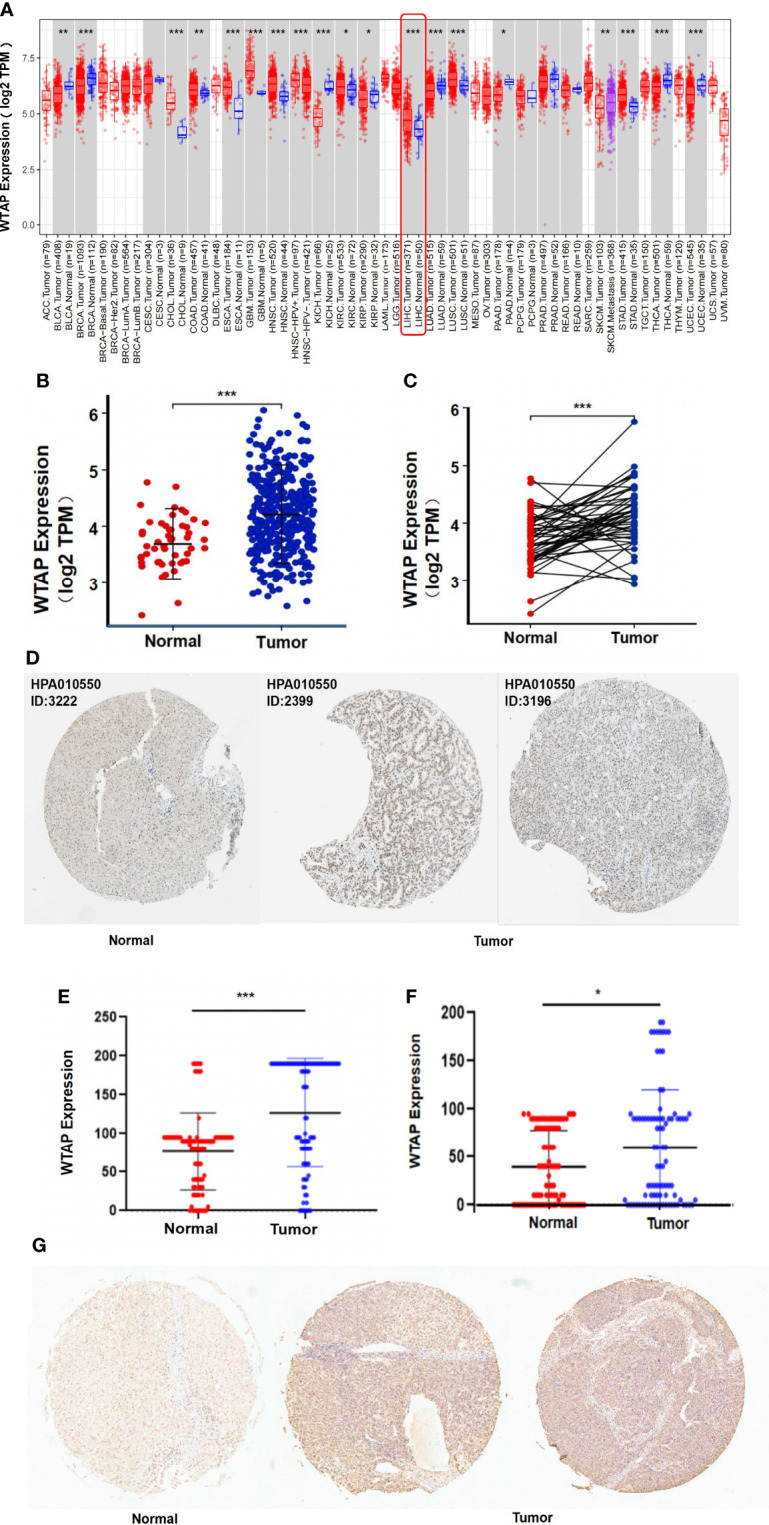
WTAP expression analysis: **(A)** Comparison between tumor tissues of 33 types of cancer and healthy samples based on the TIMER database. **(B, C)** Comparison between unpaired or paired HCC tumor tissues and healthy samples derived from the TCGA dataset. **(D)** Immunohistochemistry images from The Human Protein Atlas of normal (left) and tumor tissues (right). **(E, F)** Comparison of total scores of immunohistochemical staining of the cytoplasm or the nucleus between normal and tumor tissues. **(G)** Immunohistochemistry images from tissue microarrays of normal (left) and tumor tissues (right). Data are presented as mean ± SD.*P < 0.05, **P < 0.01, ***P < 0.001.

RNA-Seq data of various human cancers from the TIMER database were analyzed for their *WTAP* transcription level (**Figure 1A**). HCC samples presented considerably increased levels of *WTAP* mRNA compared with healthy liver samples. WTAP was also highly expressed in the tissues of the following cancer types: colon adenocarcinoma, cholangiocarcinoma, glioblastoma multiforme, head and neck squamous cell carcinoma, lung squamous cell carcinoma, esophageal carcinoma, kidney renal clear cell carcinoma, and stomach adenocarcinoma. In contrast, WTAP expression was downregulated in breast invasive carcinoma, kidney renal papillary cell carcinoma, kidney chromophobe, bladder urothelial carcinoma, pancreatic adenocarcinoma, thyroid carcinoma, lung adenocarcinoma, and uterine corpus endometrial carcinoma samples compared with that in non-tumor tissues. We conclude that WTAP is aberrantly expressed across several tumors. We then investigated WTAP expression in the TCGA datasets. The results show that WTAP was highly expressed in both unpaired and paired tumor samples with HCC (**Figure 1B, C**).

At the protein level, WTAP was expressed at moderate to high levels in HCC tissues and low level in normal liver tissues as evidenced by immunohistochemistry staining based on The Human Protein Atlas database (**Figure 1D**). To confirm the above result, we used immunohistochemical staining to detect WTAP level in 90 pairs of HCC and the adjacent normal tissue. HCC tumor tissue presented higher WTAP levels, in both cytoplasm and nucleus, compared with healthy liver samples (**Figure 1E-G**).

### WTAP Expression Is Correlated With Clinicopathological Features and Poor Prognosis

The clinicopathological characteristics of the 90 patients stratified by low or high WTAP expression are summarized in **Table 1**. We found a significant association between low cytoplasmic WTAP expression and tumor grade (*P* = 0.04). Conversely, nuclear WTAP level did not correlate with tumor grade (**Table 2**). Moreover, WTAP expression did not appear to be related to other clinical parameters such as sex, age, tumor size, T stage, and TNM stage.

**Table 1:** Relationship between WTAP expression levels in the cytoplasm and clinicopathological characteristics in HCC patients.

	Variables	WTAP expression		total	χ^2^	p value
		low	high			
Sex					2.009	0.156
	male	34	39	73		
	Female	10	5	15		
Age (year)					0.045	0.831
	≤54	23	22	45		
	>54	21	22	43		
Grade					8.250	0.004
	I/II	38	26	64		
	III	6	18	24		
Tumor size					0.727	0.394
	≤5.5cm	20	24	44		
	>5.5cm	24	20	44		
T stage					0.245	0.620
	T1/T2	26	25	51		
	T3/T4	17	13	30		
TNM stage					0.245	0.620
	I/II	26	25	51		
	III	17	13	30		

**Table 2:** Relationship between WTAP expression levels in the nucleus and clinicopathological characteristics in HCC patients.

	Variables	WTAP expression		total	χ^2^	p value
		low	high			
Sex					1.155	0.283
	male	60	13	73		
	Female	14	1	15		
Age (year)					1.152	0.283
	≤54	36	9	45		
	>54	38	5	43		
Grade					0.014	0.905
	I/II	54	10	64		
	III	20	4	24		
Tumor size					1.359	0.244
	≤5.5cm	39	5	44		
	>5.5cm	35	9	44		
T stage					1.876	0.171
	T1/T2	45	6	51		
	T3/T4	23	7	30		
TNM stage					0.152	1.369
	I/II	45	6	51		
	III/IV	23	7	30		

**Figure 2:**
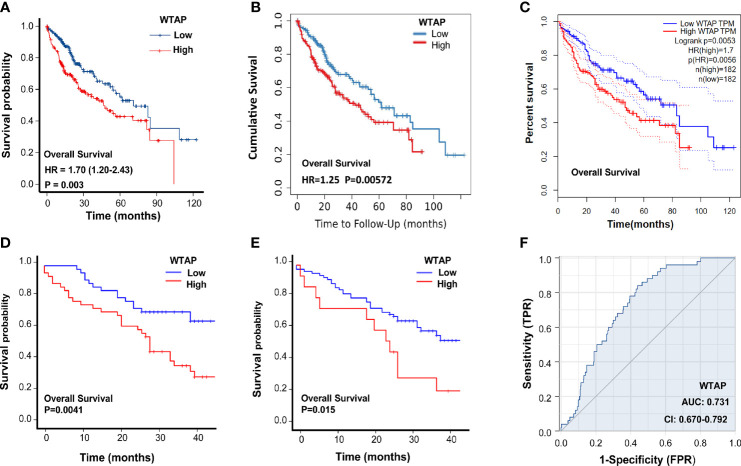
Association between WTAP expression and HCC prognosis. **(A-C)** Overall survival of patients in the WTAP high- and low-expression groups in TCGA, TIMER2.0, and GPEIA. **(D)** Overall survival of HCC patients with WTAP high- and low-expression in the cytoplasm analyzed using clinical information of tissue microarrays. **(E)** Overall survival of HCC patients with WTAP high- and low-expression in the nucleus analyzed using clinical information of tissue microarrays. **(F)** Area under the curve value of the risk signature to predict the overall survival of patients with HCC.

Next, the prognostic value of WTAP expression in HCC was investigated. As indicated in **Figure 2A**, a higher WTAP expression was significantly associated with shorter overall survival based on the TCGA data (hazard ratio = 1.70, *P* = 0.003). These results were verified using TIMER2.0 and GPEIA, and similar results were obtained (**Figures 2B, C**). We then analyzed the prognostic data of tissue microarrays. The WTAP level in the cytoplasm and nucleus negatively corelated with survival time (**Figures 2D, E**). Then, the univariate and multivariate analysis results demonstrated that the WTAP expression level is an independent predictor of unfavorable prognosis in patients with HCC (**Tables 3** and **4**). Furthermore, the result of receiver operating characteristic curves (ROC)obtained an area under the curve value of 0.731 (confidence interval: 0.670–0.792), which representing good performance of ROC curves (**Figures 2F**).

**Table 3:** Univariate and multivariate analyses of WTAP expression in the cytoplasm associated with overall survival of liver cancer patients.

variables	Univariate analysis				Multivariate analysis			
	P	HR	95%CI		P	HR	95%CI	
			Mix	Max			Mix	Max
WTAP	0.006	2.446	1.292	4.630	0.025	2.283	1.108	4.707
sex	0.059	0.369	0.131	1.037				
Age	0.125	0.619	0.336	1.142				
Grade stage	0.034	1.952	1.051	3.628	0.722	1.143	0.548	2.384
Tumor size	0.008	2.334	1.245	4.375	0.337	1.661	0.589	4.681
T stage	0.008	2.442	1.266	4.709	0.311	1.676	0.618	4.547
TNM stage	0.008	2.442	1.266	4.709	0.311	1.676	0.618	4.547

**Table 4:** Univariate and multivariate analyses of WTAP expression in the nucleus associated with overall survival of liver cancer patients.

variables	Univariate analysis				Multivariate analysis			
	P	HR	95%CI		P	HR	95%CI	
			Mix	Max			Mix	Max
WTAP	0.019	2.279	1.147	4.528	0.044	2.143	1.020	4.501
sex	0.059	0.369	0.131	1.037				
Age	0.125	0.619	0.336	1.142				
Grade stage	0.034	1.952	1.051	3.628	0.248	1.514	0.749	3.059
Tumor size	0.008	2.334	1.245	4.375	0.479	1.438	0.526	3.932
T stage	0.008	2.442	1.266	4.709	0.362	1.583	0.589	4.256
TNM stage	0.008	2.442	1.266	4.709	0.362	1.583	0.589	4.256

### Genes Co-Expressed With WTAP

**Figure 3:**
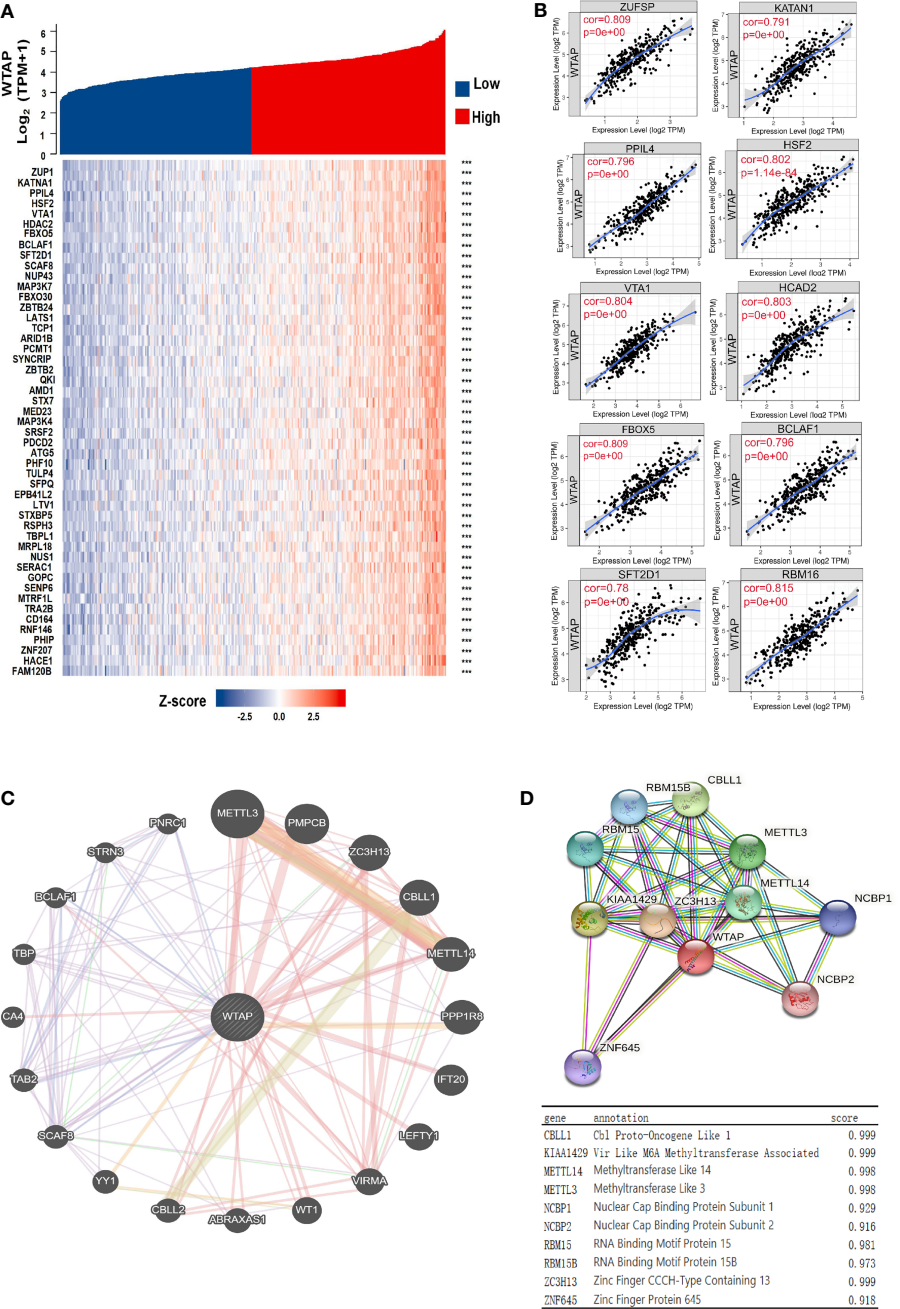
**(A)** Heatmap showing the top 50 genes most strongly related to WTAP. **(B)** Top 10 genes co-expressed with WTAP in HCC samples. **(C, D)** Proteins interacting with WTAP in HCC tissues and their co-expression scores. ***P < 0.001.

We identified the genes that were co-expressed with *WTAP* through data mining. The top 10 significant genes correlated with WTAP were TBP, PPIL4, ZUP1, VTA1, FBXO5, HSF2, KATNA1, HDAC2, FBXO30, and PHF10 (**Figure 3A**). The relationships between *WTAP* and these genes was confirmed by the TIMER database (**Figure 3B**).

### Protein Interaction Networks of WTAP Protein

To elucidate the molecular mechanism underlying HCC malignancy and its progression, we constructed the protein interaction networks of the WTAP protein (**Figure 3C**). The top 10 proteins and their corresponding gene names, annotations, and scores are shown in **Figure 3D**. These genes included CBLL1, VRIMA, METTL14, METTL3, NCBP1, NCBP2, RBM15, RBM15B, ZC3H13, and ZNF645.

### WTAP-Related Signaling Pathways in HCC

**Figure 4:**
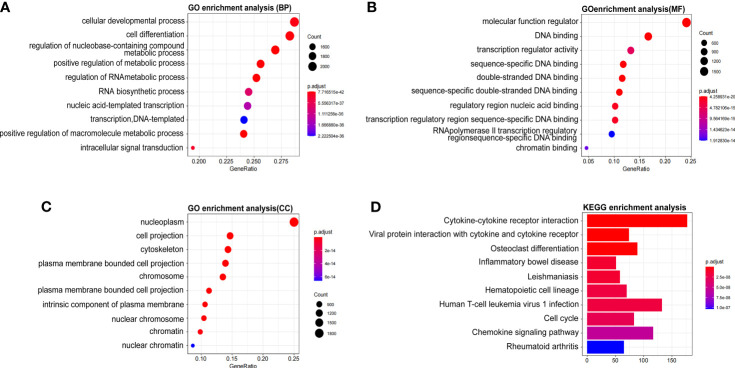
**(A–C)** Significant Gene Ontology terms outlining biological processes **(A)**, molecular functions **(B)**, and cell components **(C)** of the top 1000 genes most positively associated with WTAP. **(D)** Significant Gene Set Enrichment Analysis results of the KEGG pathways.

The molecular functions of WTAP in HCC was further analyzed using Gene Ontology and KEGG to predict WTAP-related signaling pathways. The most enriched terms were “cellular developmental process”, “molecular function regulator”, and “nucleoplasm” in the biological processes (BP), molecular functions (MF), and cellular components (CC), respectively (**Figures 4A–C**). According to the results of KEGG analysis, the most relevant signaling pathways included “cytokine–cytokine receptor interaction”, “viral protein interaction with cytokine and cytokine receptor”, and “osteoclast differentiation” (**Figure 4D**). Moreover, WTAP closely correlated with the incidence of cell cycle and immune diseases such as inflammatory bowel disease and rheumatoid arthritis.

### WTAP Correlated With the Infiltration of Immune Cells

**Figure 5:**
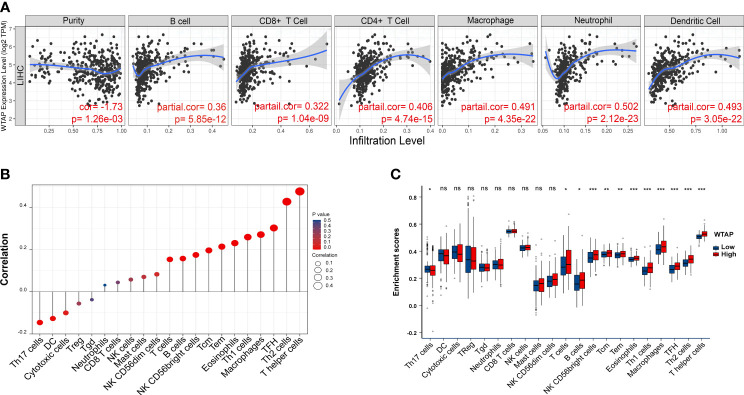
**(A)** WTAP expression is correlated with the degree of infiltration of CD4+ T cells, CD8+ T cells, macrophages, B cells, myeloid dendritic cells, and neutrophils in HCC tissues. **(B)** Correlation between the relative abundance of 21 immune cell types and WTAP expression levels. Dot sizes represent the absolute value of Spearman R. **(C)** Box diagram showing differences in degrees of infiltration of 21 immune cells between WTAP high- and low-expression groups. *P < 0.05, **P < 0.01 and ***P < 0.001 respectively. "ns" not statistically significant.

Additional analyses were performed using the TIMER database to elucidate the core action between WTAP expression and HCC immune cell infiltration. As shown in **Figure 5A**, WTAP expression strongly correlated to WTAP level of different infiltrating immune cell types, including neutrophils (*P* < 0.001), dendritic cells (*P* < 0.001), macrophages (*P* < 0.001), CD4+ T cells (< 0.001), B cells (*P* < 0.001), and CD8+ T cells (*P* < 0.001). Furthermore, the ssGSEA showed that WTAP expression positively correlated with tumor-infiltrating immune cells (**Figures 5B, C**), including T helper cells, Th2 cells, follicular helper T cells, macrophages, Th1 cells, eosinophils, central memory T cell, effective memory T cell, NK CD56 bright cells, B cells, and T cells. In contrast, WTAP expression negatively correlated with Th17 cells.

**Figure 6:**
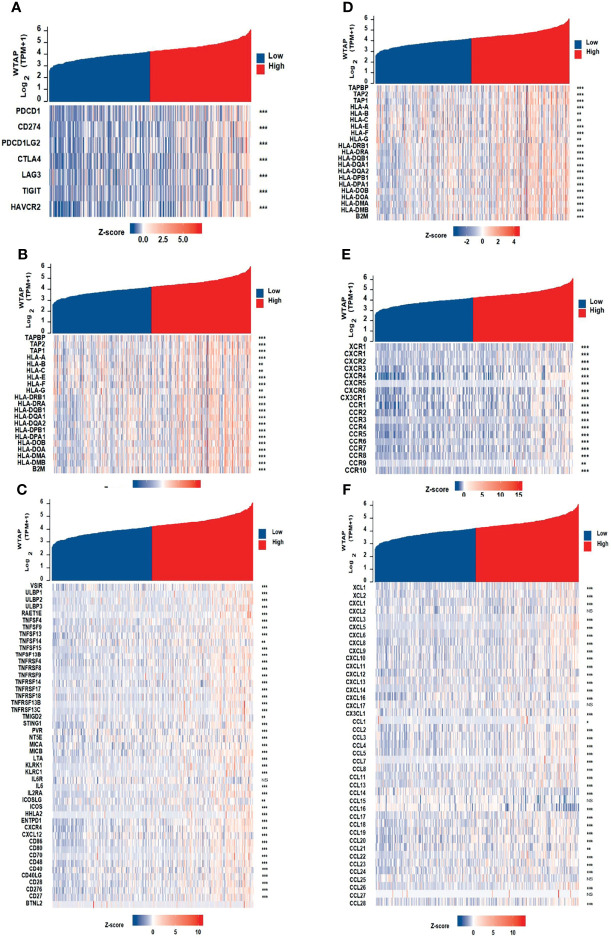
Co-expression of WTAP and immune-related genes, including immune checkpoint genes **(A)**, immunosuppressive genes **(B)**, immune activation genes **(C)**, MHC genes **(D)**, chemokines receptor genes **(E)**, and chemokines genes **(F)**.

We hypothesized that the expression of WTAP may be associated with that of immune checkpoint genes. Heatmap and correlations between WTAP and immune checkpoints, including PD-1, PD-L1, PD-L2, CTLA4, LAG3, TIGIT, and HAVCR4, are shown in **Figure 6A**. there were significant and positive correlation between all these immune checkpoint genes and WTAP expression in HCC samples from the TCGA database. Then, the gene co-expression analysis showed that the expression of WTAP correlated with that of genes encoding immunosuppressive genes, immune activation genes, major histocompatibility complexes genes, chemokines receptor genes, and chemokines genes (**Figures 6B–F**). The resulting heatmap showed that many immune-related genes were co-expressed with WTAP, and the majority were positively correlated with WTAP, except for KIR2DL1, IL6R, CXCL2, CXCL17, CCL15, CCL25, and CCL27.

### WTAP Knockdown Inhibits HCC Proliferation and Invasion *In Vitro*

**Figure 7:**
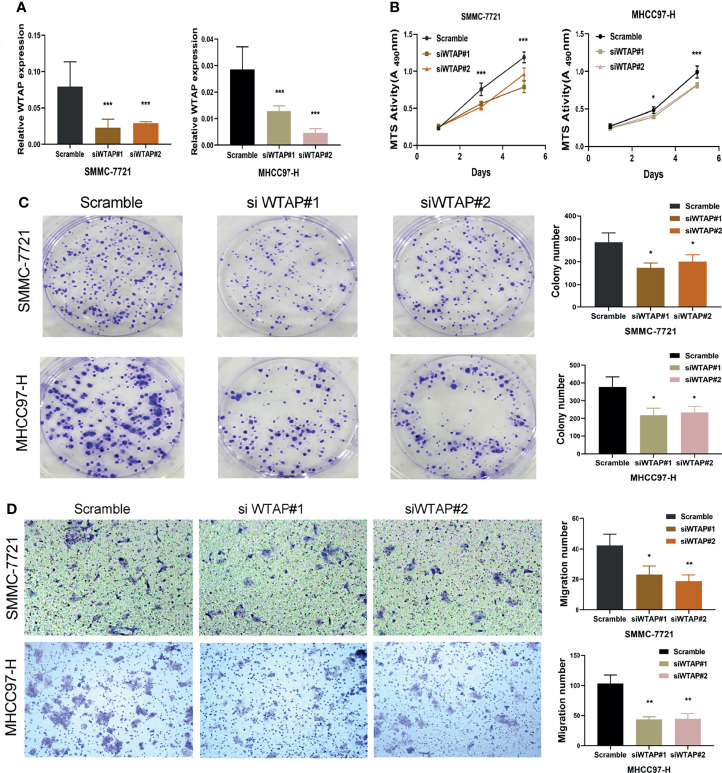
Knockdown of WTAP inhibited the proliferation and migration of hepatocellular carcinoma cells. **(A)** SMMC-7721 and MHCC97-H cells were transfected with si-WTAP, and WTAP levels were evaluated by qRT-PCR. **(B, C)** Proliferation of SMMC-7721 and MHCC97-H cells was examined using MTS and colony formation assays. **(D)** Migration of hepatocellular carcinoma cells was examined using Transwell assays. *P < 0.05, **P < 0.01 and ***P < 0.001 respectively.

Transfection efficiency was examined (**Figure 7A**). WTAP knockdown considerably suppressed SMMC-7721 and MHCC97-H cell proliferation (**Figures 7B, C**) as evidenced by MTS and colony formation assay results. WTAP knockdown also considerably reduced HCC cell invasion and migration (**Figure 7D**).

## Discussion

In the present study, dry and wet experiments were utilized to uncover detailed information on WTAP. Initially, bioinformatics analysis shows that WTAP expression was considerably increased in HCC tissues and was associated with shorter overall survival, suggesting that WTAP likely functions as an oncogene that promotes liver carcinogenesis (**Figures 1**, **2**). These findings are consistent with a previous study that reported a higher WTAP expression in HCC tumor tissues than in healthy liver samples, and it appears to be significantly correlated with patient clinical stage and prognosis (21).

Then, the genetic correlation of WATP was investigated (**Figure 3**). WTAP was co-expressed with the following genes: TBP, PPIL4, ZUP1, VTA1, FBXO5, HSF2, KATNA1, HDAC2, FBXO30, and PHF10 (**Figures 3A, B**). Both PPI network analyses by GeneMANIA and The Search Tool for the Retrieval of Interacting Genes/Proteins website highlighted a strong correlation between WTAP and CBLL1, VRIMA, METTL14, METTL3, NCBP1, NCBP2, RBM15, RBM15B, ZC3H13, and ZNF645, all of which are primarily involved in m6A modification (**Figures 3C, D**). As “writers” of the m6A modification, METTL3, METTL14, KIAA1429, and RBM15 were identified as important proteins interacting with WTAP in HCC. Meanwhile, using KEGG pathway analysis (**Figure 4D**), WTAP was found to be linked to cytokine–cytokine receptor interaction pathways, influencing the cell cycle. WTAP has also been considered a regulator of cell cycle in another study (22). These results indicate that the overexpression of WTAP may be the mechanism underlying the functional imbalance of m6A modification as a negative predictor of HCC.

Immune cell infiltration was shown to play important roles in tumor initiation and progression (23). However, there is a complex relationship between HCC development and the intrinsic role of the liver in the immune system. On one hand, owing to the exposure to low-dose endotoxins and food antigens from the intestine, the liver is physiologically programmed to be immunotolerant to foreign, gut-derived particles (24). On the other hand, the liver forms a critical component of the body’s immune response, enriching Kupffer cells, liver endothelial cells, and innate immune cells. In addition, the inflammatory activation of hepatic stellate and Kupffer cells mediates immune cell infiltration *via* chemokines (25). Because the vital role of the immune system in HCC, the relationship between HCC immune cell infiltration and WTAP should be elucidated. In this work, immune analysis elucidated a positive correlation between WTAP expression and the infiltration levels of neutrophils, dendritic cells, macrophages, CD4+ T cells, B cells, and CD8+ T cells in HCC tissues (**Figure 5A**). It was found that the expression of WTAP was related to that of immune checkpoint genes (**Figure 5B**). PD-1, CTLA4, LAG3, TIGIT, and TIM-3 (HAVCR2) are known as inhibitory molecules expressed in exhausted T cells that have an impaired ability to kill tumors because they neither respond to T cell receptor stimulation nor secrete anti-tumor cytokines such as interferon γ and tumor necrosis factor-α (26). Furthermore, we explored the association between the level of WTAP and that of other immune-related genes in HCC using gene co-expression analyses and revealed the co-expression of WTAP with genes encoding major histocompatibility complexes, immune activation molecules, immunosuppressive molecules, chemokines, and chemokine receptor proteins (**Figure 6**).These results revealed the oncogenic role of WTAP and the link between its expression and the degree of tumor immune cell infiltration in human HCC tissues, thus providing a deeper understanding of the potential role of WTAP in HCC immunology and its prognostic value.

Finally, we preliminarily demonstrated the cancer-promoting effect of WTAP in tumor cell lines (**Figure 7**). The silencing of the WATP gene significantly inhibited the proliferation and migration of tumor cell lines, which further confirmed the important role of WTAP in the tumorigenesis and progression of HCC. Nevertheless, the current study has not been able to elucidate the specific mechanism by which WTAP acts as an oncogene and further research needs to conduct. There is still a long way to fully reveal the regulatory mechanism of WTAP in HCC. Relevant molecular biology experiments and *in vivo* analysis will be the focus of our future research.

## Conclusion

WTAP was expressed at elevated level in HCC tissues compared with that in normal tissues. It was associated with a worse prognosis of patients and the immune infiltration of tumor cells. Furthermore, WTAP knockdown inhibited HCC proliferation and aggressiveness. Thus, WTAP may play a pathogenic role in HCC progression by acting on both tumor cells and tumor-infiltrating immune cells. Our study indicates the potential role of WTAP as a biological marker for HCC prognosis.

## Data Availability Statement

The datasets presented in this study can be found in online repositories. The names of the repository/repositories and accession number(s) can be found in the article/**Supplementary Material**.

## Ethics Statement

The studies involving human participants were reviewed and approved by Ethics Committee of Shanghai Xinchao Technology Co., Ltd. The patients/participants provided their written informed consent to participate in this study.

## Author Contributions

YL and QY designed this study. QD and LQ extracted the information from the databases. LJL and HX analyzed the data. LGL, WZ and YL supervised the entire study. LJL wrote the manuscript. All authors contributed to the article and approved the submitted version.

## Funding

This study was supported by the National Key Research and Development Program of China (No. 2017YFA0205200), the National Natural Science Foundation of China (No. 62027901, No. 81901857, No. 81902460), the Natural Science Foundation of Guangdong Province, China (No.2022B1515020010), the Guangdong Provincial Key Laboratory of Tumor Interventional Diagnosis and Treatment (No. 2021B1212040004) and the Science and Technology Development Fund, Macau SAR (No. 0011/2019/AKP).

## Conflict of Interest

The authors declare that the research was conducted in the absence of any commercial or financial relationships that could be construed as a potential conflict of interest.

## Publisher’s Note

All claims expressed in this article are solely those of the authors and do not necessarily represent those of their affiliated organizations, or those of the publisher, the editors and the reviewers. Any product that may be evaluated in this article, or claim that may be made by its manufacturer, is not guaranteed or endorsed by the publisher.
