# Supplementary material for: WTAP Is Correlated With Unfavorable Prognosis, Tumor Cell Proliferation, and Immune Infiltration in Hepatocellular Carcinoma
Source: Front Oncol. 2022 Apr 11;12:852000. doi: 10.3389/fonc.2022.852000 (PMC9035869; doi:10.3389/fonc.2022.852000)
Supplement: Supplementary file 1 [file DataSheet_1.zip › ID85200-Supplementary material/Fig 1A-gene expression.docx]

https://cistrome.shinyapps.io/timer/
