# Supplementary material for: WTAP Is Correlated With Unfavorable Prognosis, Tumor Cell Proliferation, and Immune Infiltration in Hepatocellular Carcinoma
Source: Front Oncol. 2022 Apr 11;12:852000. doi: 10.3389/fonc.2022.852000 (PMC9035869; doi:10.3389/fonc.2022.852000)
Supplement: Supplementary file 1 [file DataSheet_1.zip › ID85200-Supplementary material/Ethical approval of tissue micrarry.pdf]

	上海芯超生物科技有限公司<br>Shanghai Outdo Biotech Company	Barcode	
	伦理委员会审核意见书	编号/Ctl No.	YB M-05-02
		版本/Version	1.0

项目名称	肝癌组织芯片产品开发及应用		
项目来源	上海芯超生物科技有限公司	项目承担单位	上海芯超生物科技有限公司
项目编号	HLivH180Su18	项目负责人	朱诗樟

审核结果

参加伦理委员会会议 7 人，投票结果：

A） 同意 7 票
B） 修正后再审查 ____ 票
C） 不同意 ____ 票
D） 暂停或终止 ____ 票

伦理委员会审查意见：

申报内容经审查，符合伦理要求，同意通过审查。

记录人签名：

陈红[illegible]

2018年 1月 8日

主任委员签名：
（伦理委员会盖章）

上海芯超生物科技有限公司 伦理委员会

年 月 日

保存期限：长期

	**上海芯超生物科技有限公司**<br>Shanghai Outdo Biotech Company	Barcode	
	样本使用伦理审查申请表	编号/Ctl No.	YB M-05-01
		版本/Version	1.0

项目名称：	肝癌组织芯片产品开发及应用	项目编号：	HLivH180Su18
项目来源：	上海芯超生物科技有限公司	资金来源：	上海芯超生物科技有限公司
样本名称：	肝癌石蜡样本	样本数量：	癌 90 例/癌旁 90 例
申请人：	沈晓静	联系方式：	021-51320288-5219
研究者 1：	高校、医院、研究所	研究者 2：	企业及其他研发机构
项目承担单位：	上海芯超生物科技有限公司		
项目负责人：	朱诗樟	联系方式：	021-51320288-5219
拟研究时间：	__2018__年__1__月__5_日 至 __2099__年__12__月_31___日		

一、 请阐述该样本用于研究的描述（包括目标和假设）：

生存期肝细胞癌 90 例——癌/癌旁各 1 点。手术时间 2010.1－2011.9，随访时间 2013.9。随访 2－3.7 年。

二、 项目研究的方法：

免疫组化、原位杂交、原位 PCR 等分子病理学实验

备注	
	申报部门意见：<br><br>部门负责人签（章）： [illegible]
说明	为有效利用宝贵的样本资源，您需向样本库提供一份简要的研究设计（如上）和所要求的资料，您要保证您将合法地使用样本，并进行您的上述研究，您需承诺在您的研究成果和文章上注明样本来源于：上海芯超•生物样本库<br><br>申请程序：下载申请表----填写申请表----负责人签字（盖章）----样本库伦理委员会审核

申请人：<br><br>[illegible]<br><br>2018 年 1 月 5 日	样本库审核意见：<br><br>样本库负责人签字： 杜莉利<br><br>2018 年 1 月 5 日

保存期限：长期
