# Supplementary figures and images for: WTAP Is Correlated With Unfavorable Prognosis, Tumor Cell Proliferation, and Immune Infiltration in Hepatocellular Carcinoma
Source: Front Oncol. 2022 Apr 11;12:852000. doi: 10.3389/fonc.2022.852000 (PMC9035869; doi:10.3389/fonc.2022.852000)

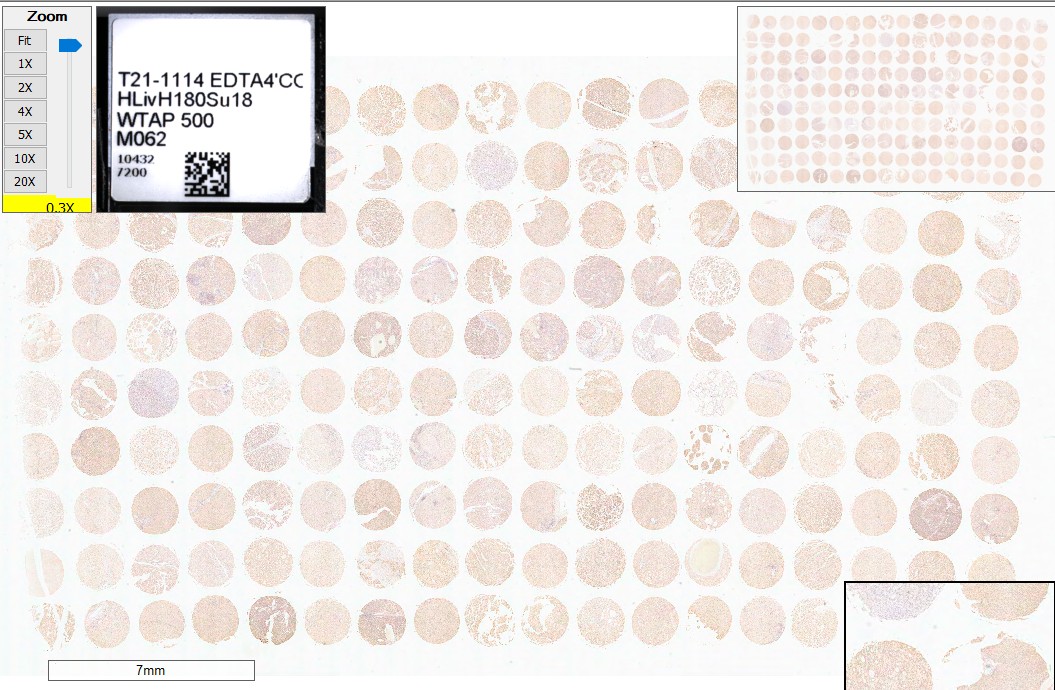

Supplement: Supplementary file 1 [file DataSheet_1.zip › ID85200-Supplementary material/Fig1-scan of tissue microarray(1).jpg]

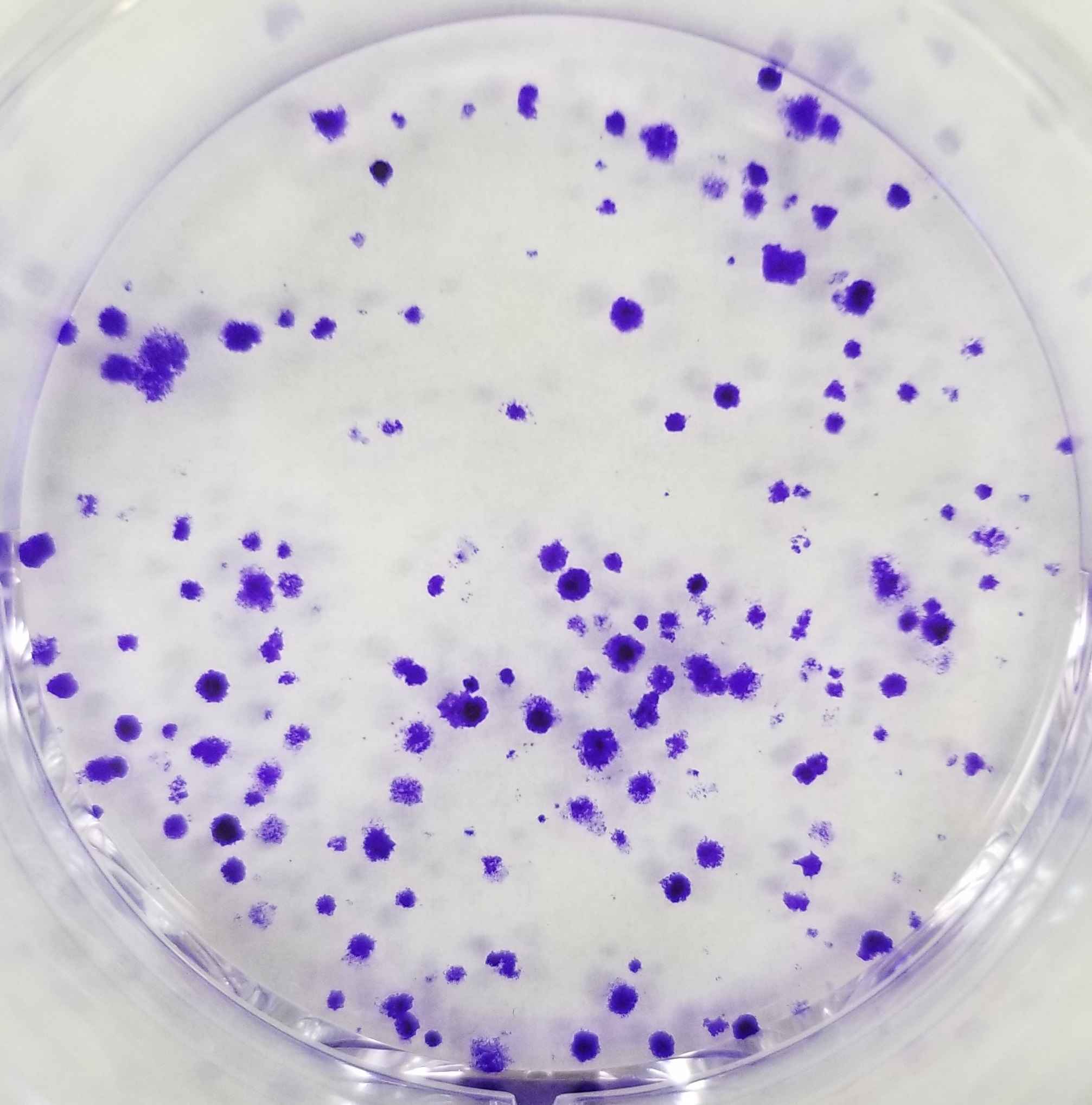

Supplement: Supplementary file 1 [file DataSheet_1.zip › ID85200-Supplementary material/Fig7C/MHCC-97 siwtap#1.jpg]

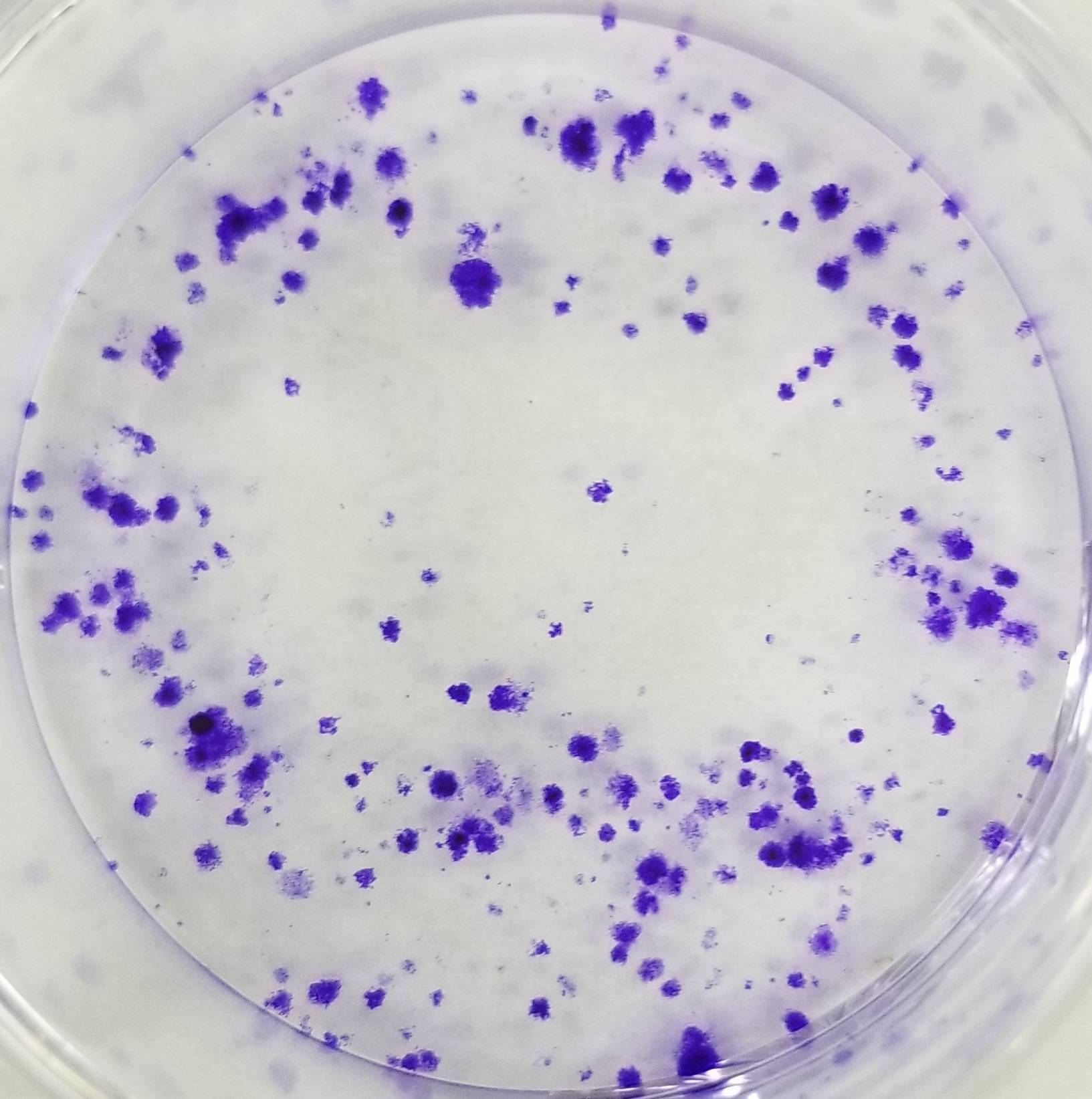

Supplement: Supplementary file 1 [file DataSheet_1.zip › ID85200-Supplementary material/Fig7C/MHCC-97 siwtap#2.jpg]

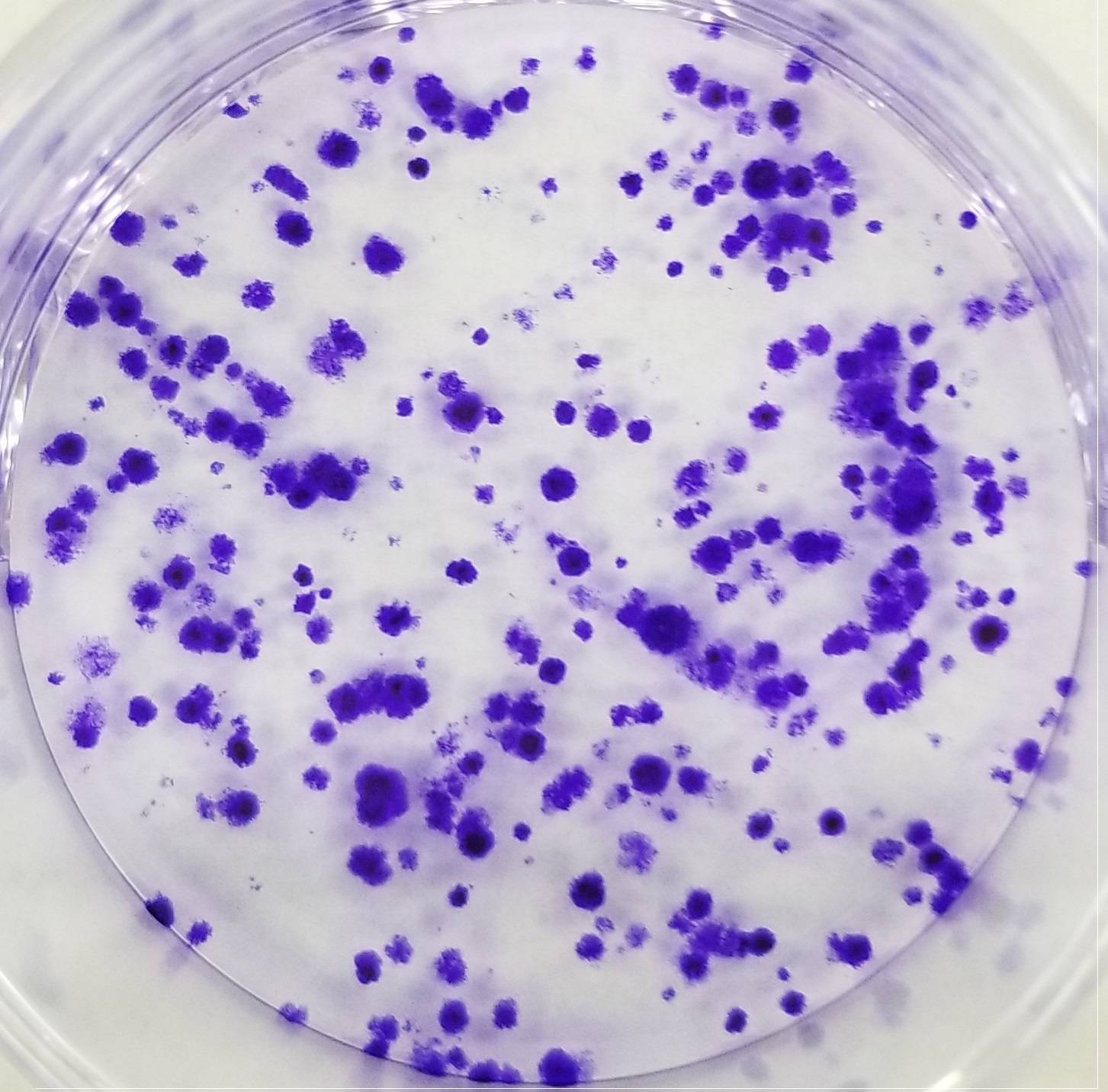

Supplement: Supplementary file 1 [file DataSheet_1.zip › ID85200-Supplementary material/Fig7C/MHCC-97H Scr.jpg]

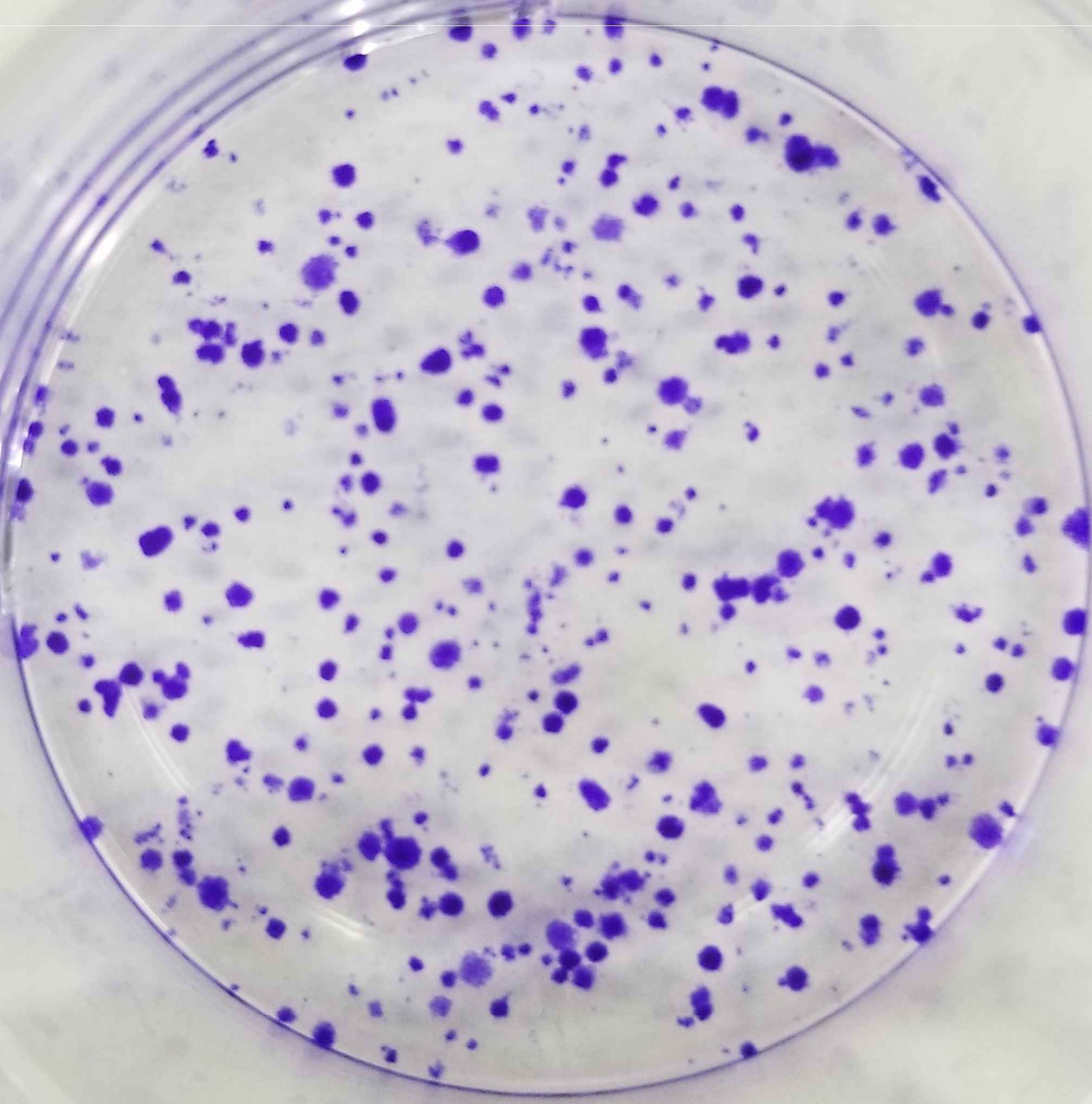

Supplement: Supplementary file 1 [file DataSheet_1.zip › ID85200-Supplementary material/Fig7C/SMMC-7721 Scr.jpg]

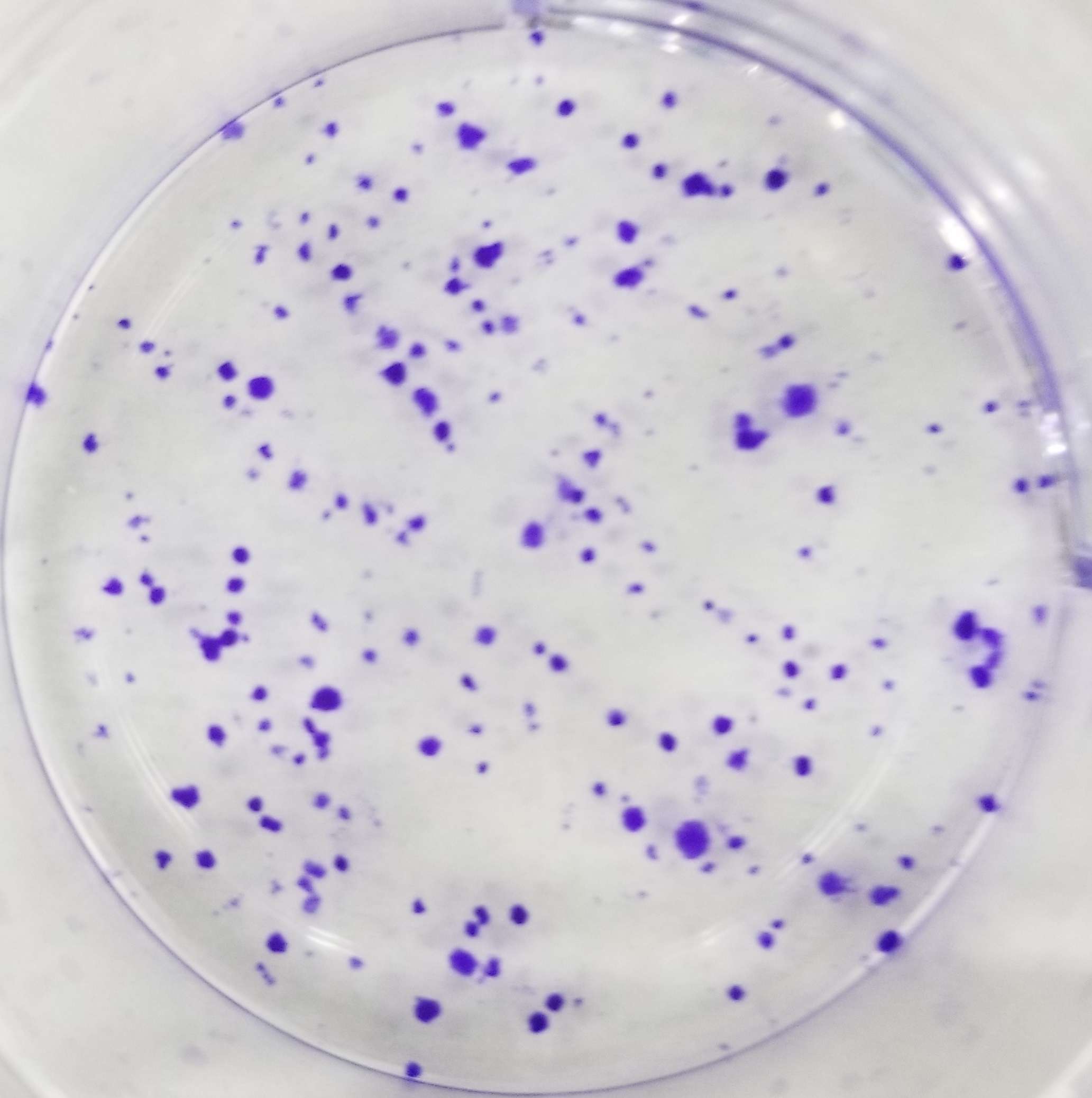

Supplement: Supplementary file 1 [file DataSheet_1.zip › ID85200-Supplementary material/Fig7C/SMMC-7721 siwtap#1.jpg]

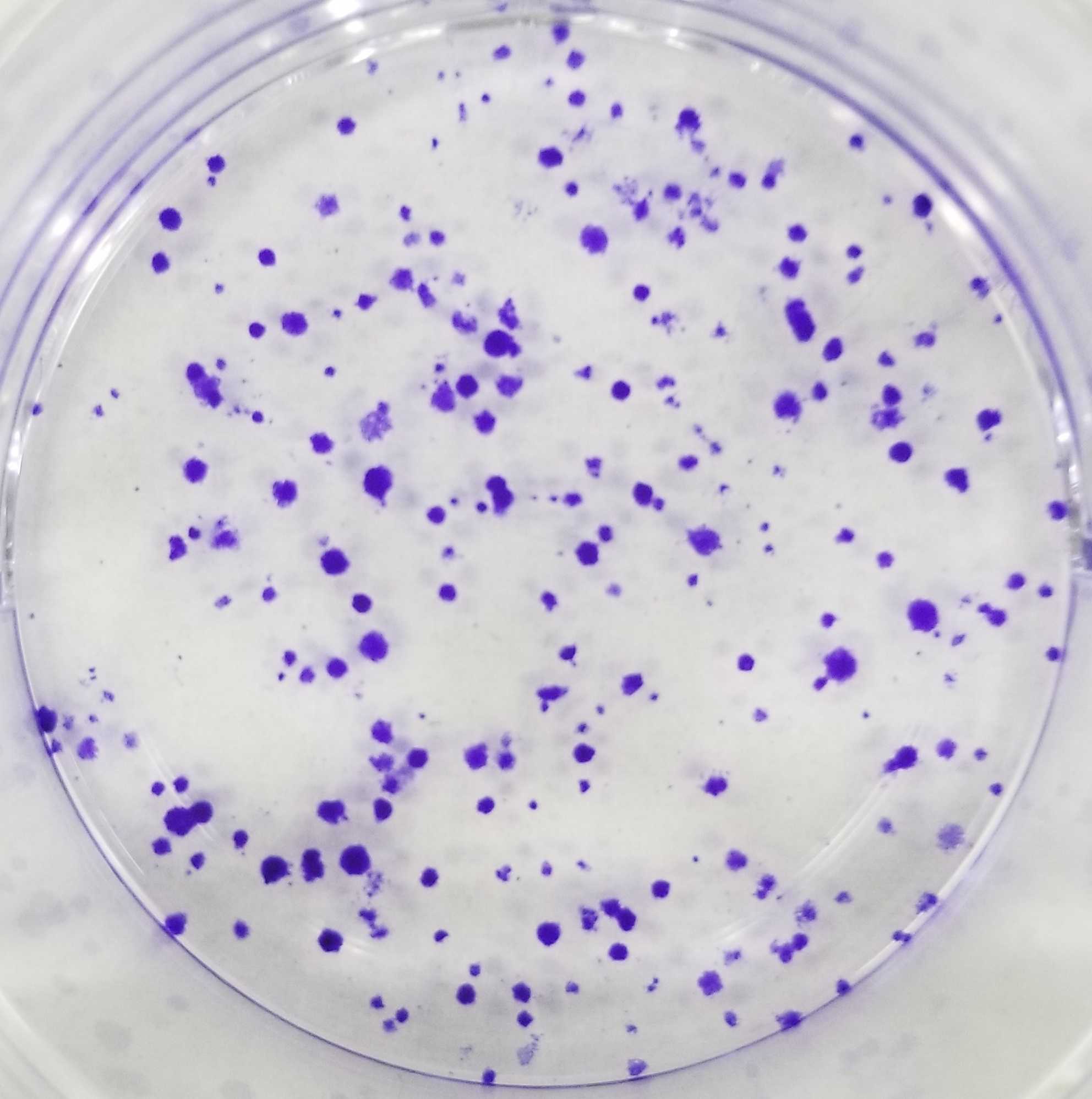

Supplement: Supplementary file 1 [file DataSheet_1.zip › ID85200-Supplementary material/Fig7C/SMMC-7721 siwtap#2.jpg]

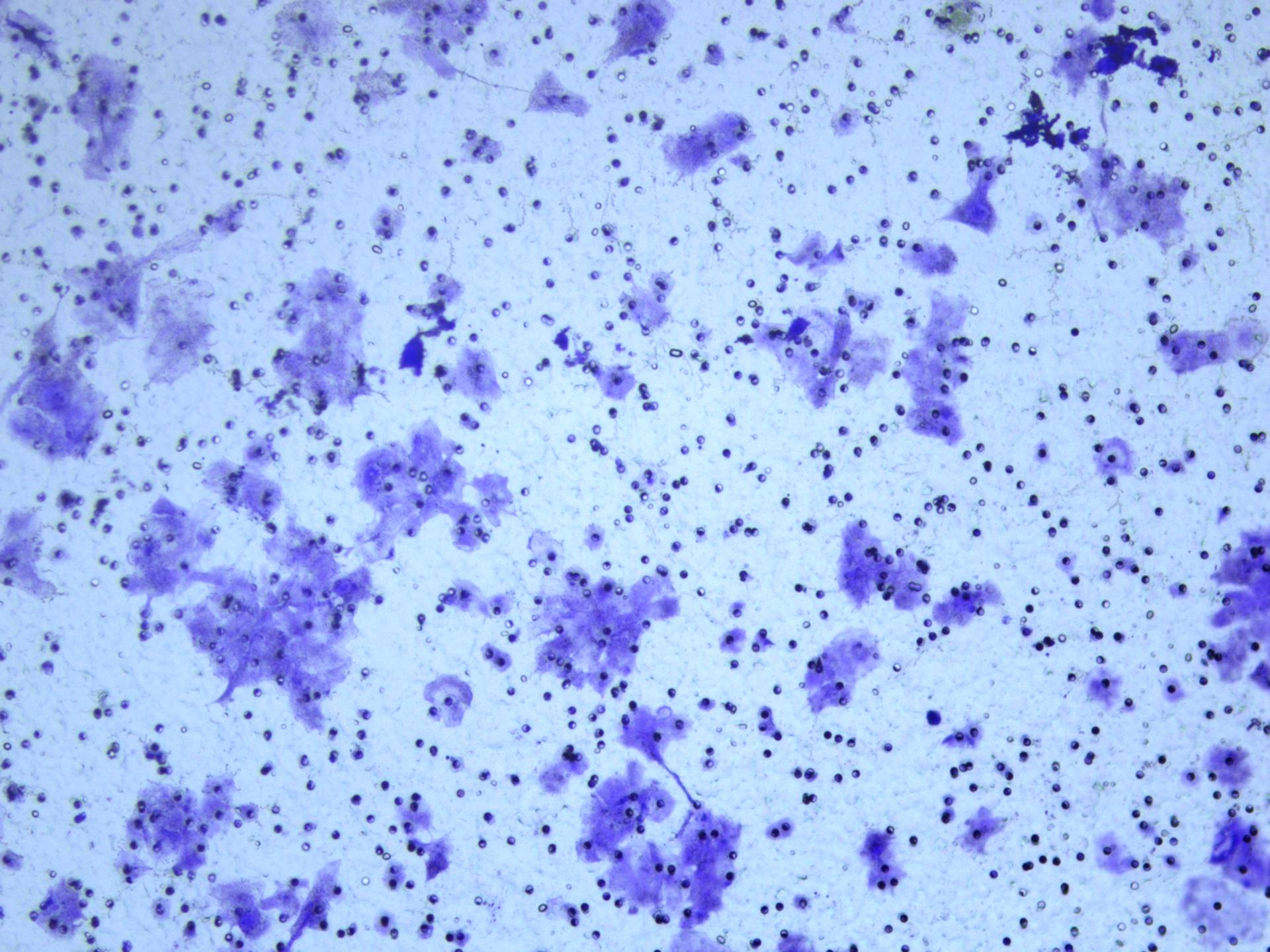

Supplement: Supplementary file 1 [file DataSheet_1.zip › ID85200-Supplementary material/Fig7D/MHCC-97H Scr.jpg]

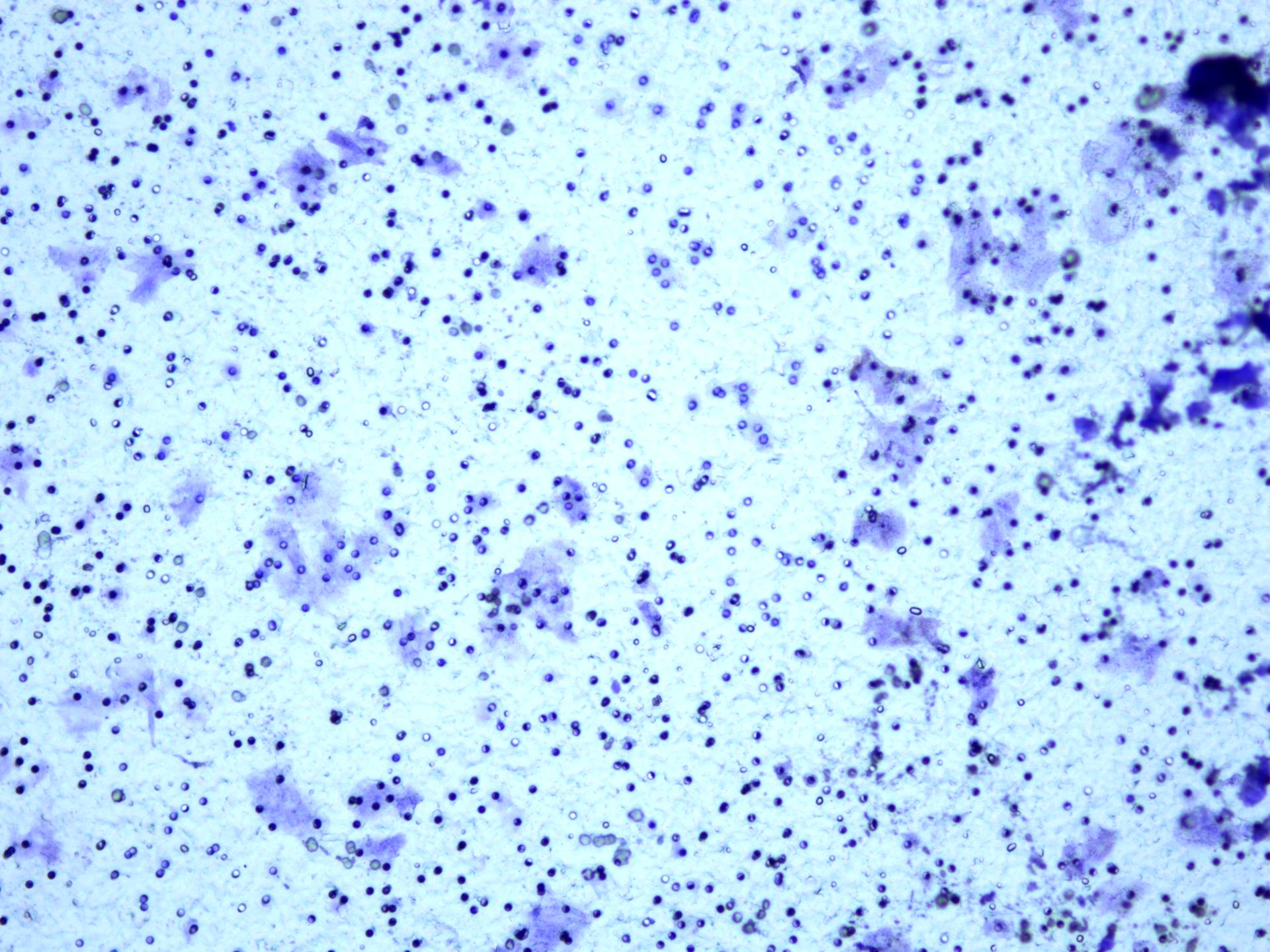

Supplement: Supplementary file 1 [file DataSheet_1.zip › ID85200-Supplementary material/Fig7D/MHCC-97H siwtap#1.jpg]

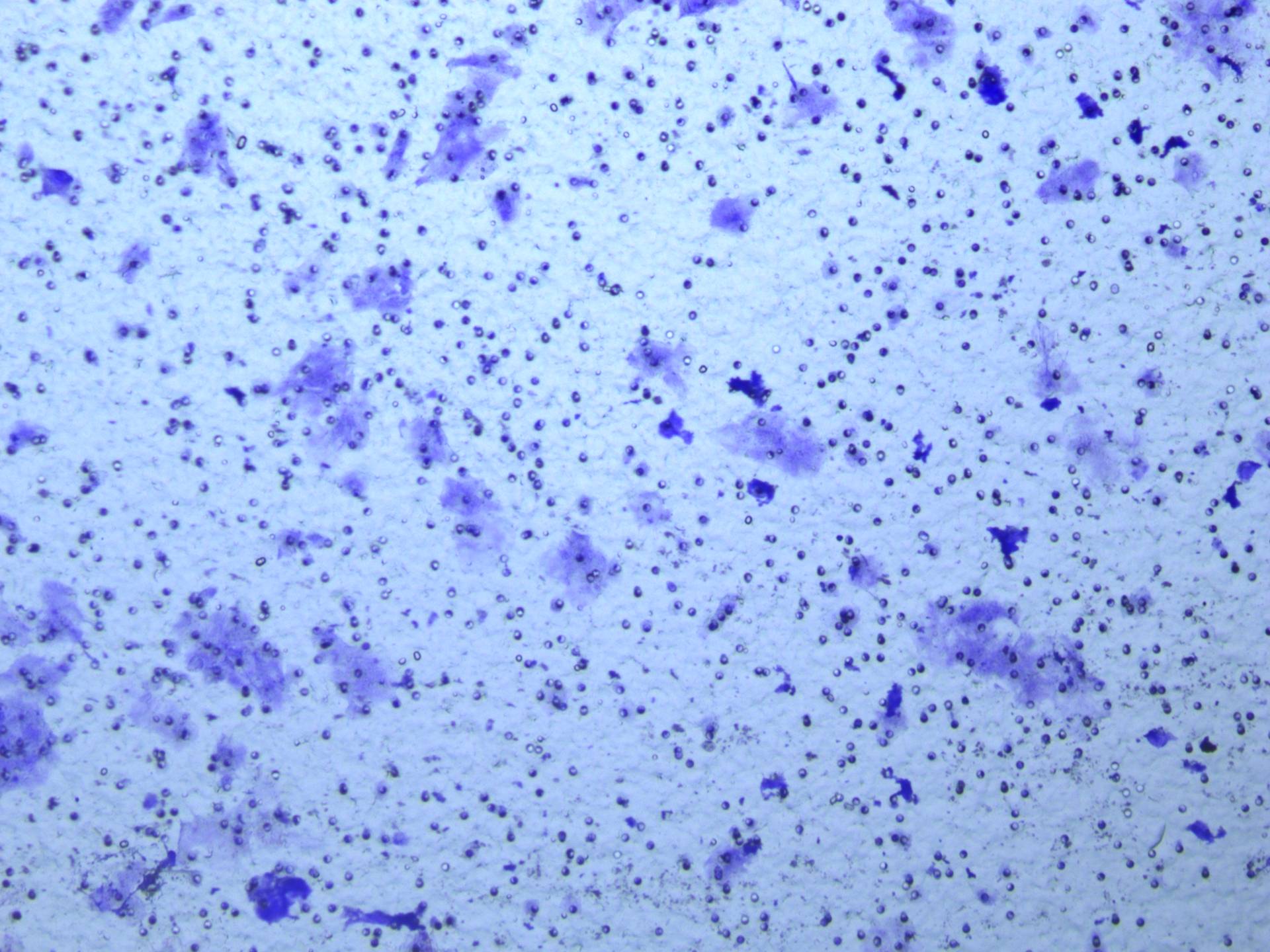

Supplement: Supplementary file 1 [file DataSheet_1.zip › ID85200-Supplementary material/Fig7D/MHCC-97H siwtap#2.jpg]

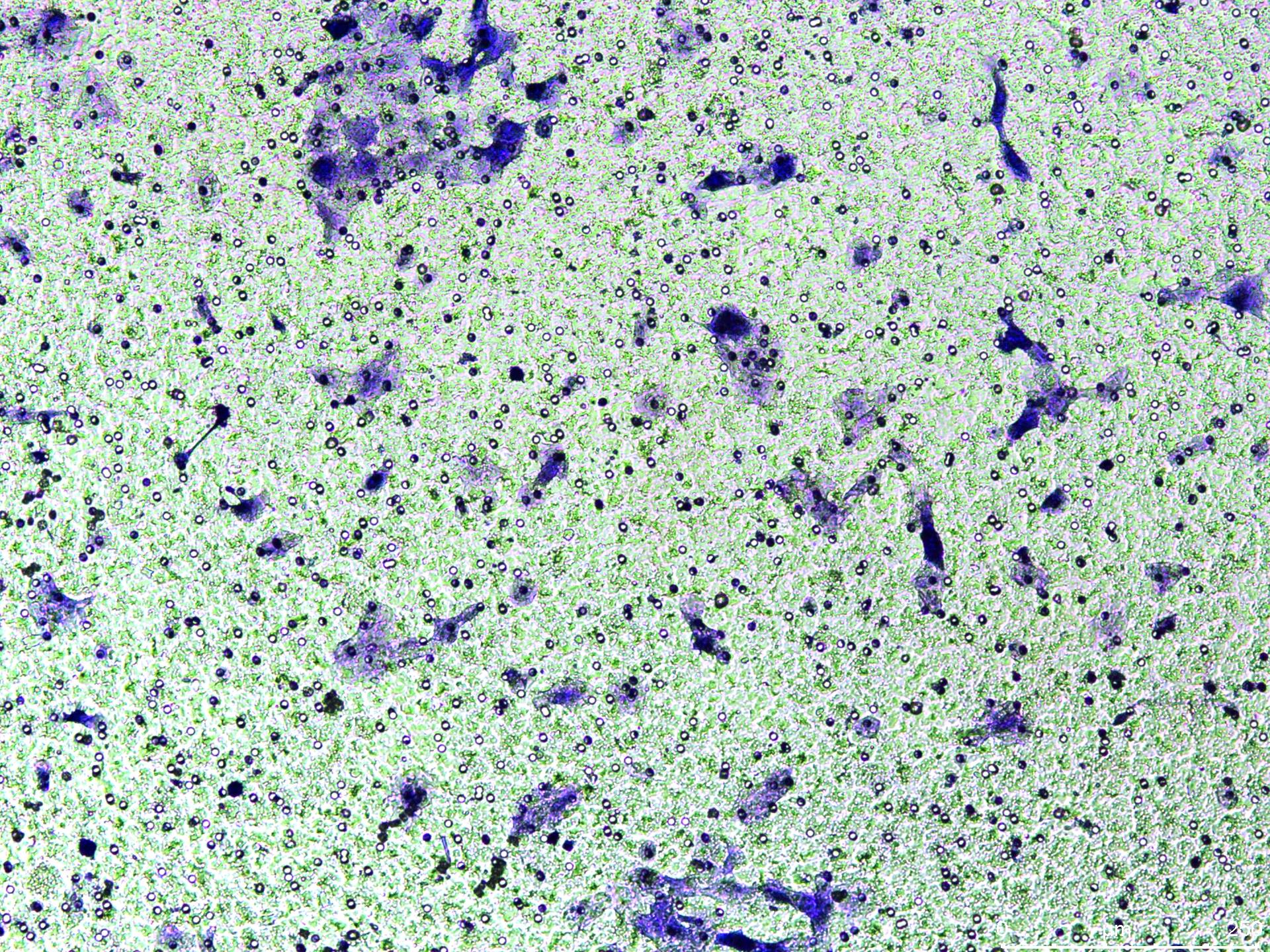

Supplement: Supplementary file 1 [file DataSheet_1.zip › ID85200-Supplementary material/Fig7D/SMCC-7721 Scr.jpg]

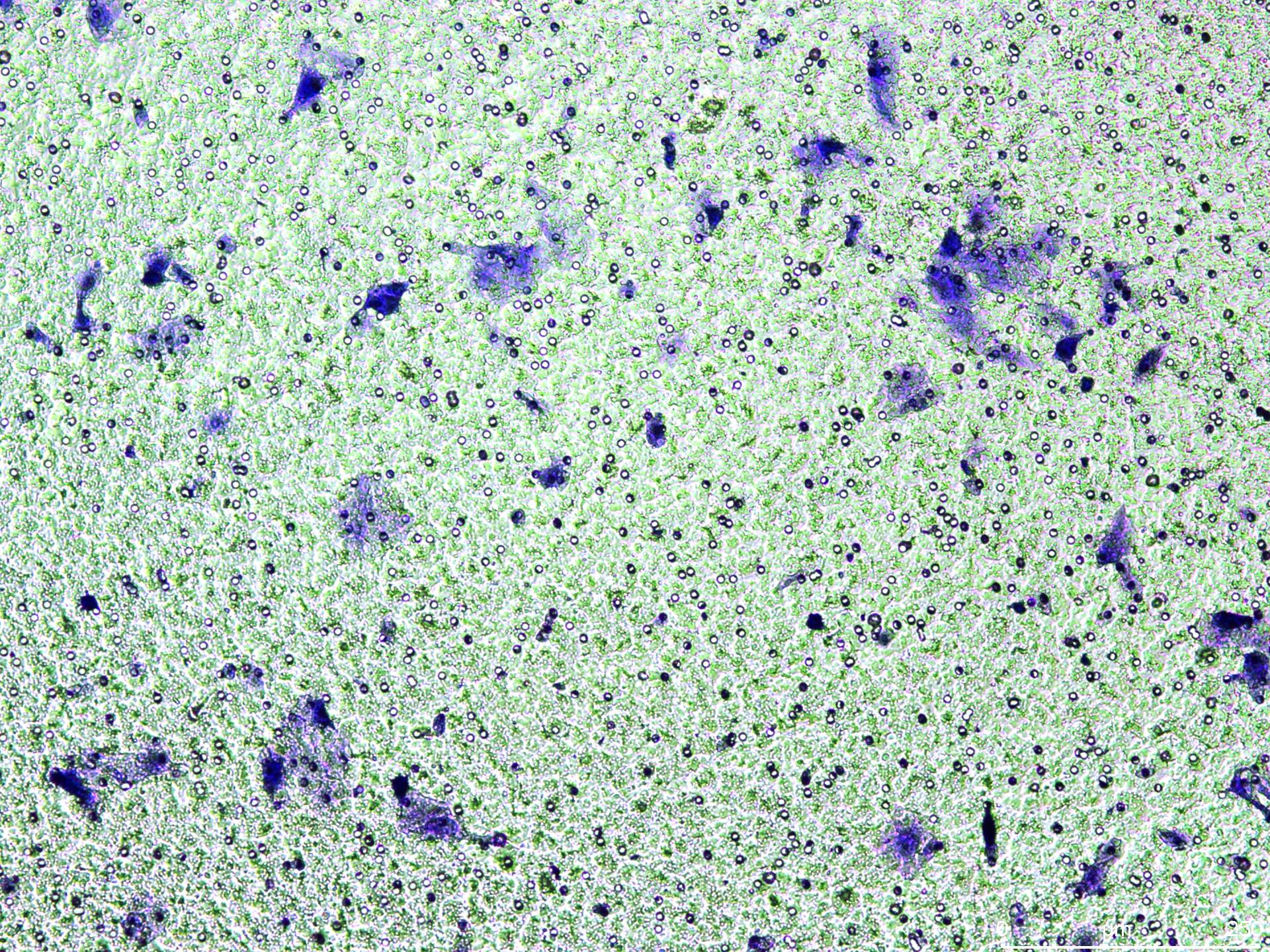

Supplement: Supplementary file 1 [file DataSheet_1.zip › ID85200-Supplementary material/Fig7D/SMCC-7721siwtap#1.jpg]

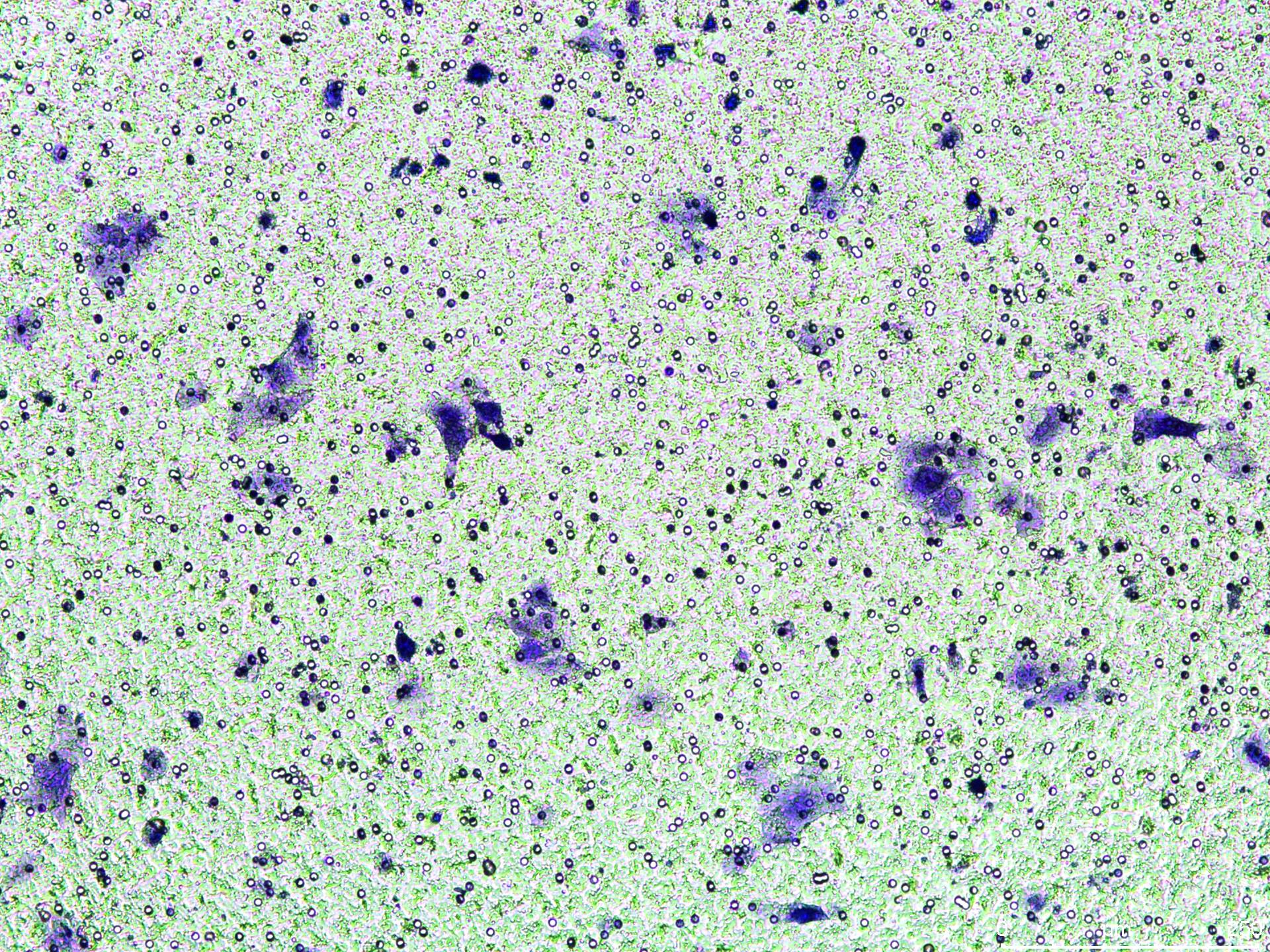

Supplement: Supplementary file 1 [file DataSheet_1.zip › ID85200-Supplementary material/Fig7D/SMCC-7721siwtap#2.jpg]
